# Psychometric Evaluation of the Polish Version of the Mindfulness Inventory for Sport (MIS-PL)

**DOI:** 10.5114/jhk/190572

**Published:** 2024-05-17

**Authors:** Zuzanna Wałach-Biśta, Dagna Kocur, Krzysztof Sas-Nowosielski

**Affiliations:** 1 Department of Pedagogy, Psychology and Sociology, Jerzy Kukuczka Academy of Physical Education in Katowice, Katowice, Poland.; 2 Institute of Psychology, University of Silesia, Katowice, Poland.

**Keywords:** questionnaire, reliability, awareness, validity

## Abstract

The study investigated psychometric properties of the Polish version of the Mindfulness Inventory for Sport (MIS-PL). Following an expert review (stage 1), a sample of 333 athletes voluntarily participated in the study to verify the internal structure of the questionnaire (stage 2). In a final step (stage 3), confirmatory factor analysis and correlation analysis of the MIS-PL subscales with other variables were conducted to demonstrate the stability and reproducibility of the factor structure of the scale and to determine the theoretical validity of the measure. The factor analyses performed confirmed the three-factor structure of the MIS-PL with the following dimensions: 1) awareness, 2) non-judgmental attitude, and 3) refocusing, characterized by acceptable fit indices. Analyses of the correlations between the MIS-PL subscales and conceptually related variables (mindfulness in daily life, worry, concentration disruption, mental toughness, and flow) also proved statistically significant. In summary, the MIS-PL is a valid measure for assessing mindfulness in sport.

## Introduction

Mindfulness, characterized by the conscious focus on present-moment experiences without judgment, has emerged as a burgeoning area of study in psychology (Bunjak et al., 2022). Extensive research has underscored the positive impacts of mindfulness-based interventions (MBIs) across diverse domains such as anxiety, depression, distress, and overall quality of life (Khoury et al., 2015). Notably, recent studies have shed light on the transformative effects of MBIs on athletes, improving athletic performance (Bühlmayer et al., 2017), fostering well-being (Foster and Chow, 2020), preventing burnout (Li et al., 2019), and reducing competitive anxiety (Ong and Chua, 2021).

Despite these advancements, existing mindfulness questionnaires have predominantly been tailored for everyday contexts and may not fully capture the nuances of mindfulness in athletic competition. Notably, they may underemphasize crucial aspects such as attentional self-regulation, which is a pivotal factor influencing sports performance (Thienot et al., 2014). Consequently, there arose a need for a tailored measure that would account for the unique application of mindfulness skills in sports settings to validate the efficacy of MBIs among athletes.

The Mindfulness Inventory for Sport (MIS) by Thienot et al. (2014) is rooted in the Gardner and Moore's (2004) Mindfulness-Acceptance-Commitment (MAC) model, which advocates for cultivating non-judgmental awareness of the present moment, embracing internal states, and sharpening focus on task-relevant cues to propel athletes towards their goals. These core principles are reflected in the MIS's factor structure: 1) awareness: adept observation of internal experiences in real-time; 2) non-judgmental attitude: accepting internal experiences without criticism; and 3) refocusing: swiftly redirecting attention to task-related stimuli. The integration of the MAC model into the MIS underscores its comprehensive approach to assessing mindfulness skills tailored specifically for athletes in sports settings.

Despite the adaptation of the Mindfulness Inventory for Sport (MIS) inventory into various languages, such as German (Wieczorek et al., 2022), Turkish (Bayköse and Çelik, 2021), Arabic (Ben Salha et al., 2022), Spanish (Moreno-Murcia et al., 2019), Persian (Hemayat Talab et al., 2016; Kashani et al., 2017), the lack of a Polish version highlights a significant research gap. Our study was designed to fill this void by conducting a rigorous evaluation of the psychometric properties of the MIS in Polish. By doing so, not only will it be possible to expand the cross-cultural utility of the MIS, but we will also be able to enrich our understanding of the role of mindfulness in the context of sports performance. The MIS can serve as a valuable tool in both the scientific exploration of sports psychology and the practical endeavors of coaches and athletes, offering insights that empower athletes to enhance their mindfulness skills, thereby improving focus, emotion regulation, and overall performance.

## Methods

### Design and Procedures

The present study consisted of three steps. The first step involved the translation and adaptation of the MIS into Polish. The second and third steps involved validation studies, involving assessment of the tool’s theoretical and criterion-based reliability and validity.

## STAGE 1

After obtaining the authors’ permission to have the original tool translated, the first step in preparing the Polish version of the MIS involved having the scale translated into Polish by three independent translators. Ten independent, competent judges then assessed the accuracy of the translations of the items with the original version. The competent judges were selected on the basis of two criteria: 1) proficiency in English, including specialized language in the field of sport psychology, 2) experience in working with mindfulness in sports. Each of the expert judges assessed three different translations of each element, with the possibility to make any corrections to the translations presented. Based on the selection of the competent judges, a list of questionnaire items was drawn up and subjected to a back-translation procedure to ensure that all questionnaire statements had been translated correctly and accurately. The preliminary version of the scale thus prepared, in which the face equivalence consisting in the correspondence between the original and the analyzed version of the questionnaire in terms of the response categories was maintained, was subjected to the bilingual response method. The psychometric equivalence of the Polish version with the original was estimated by determining the strength of correlations of all the subscales in both versions, completed by athletes proficient in English (n = 8). The results obtained (Spearman’s rho = 0.785, *p* < 0.05 for the awareness subscale; Spearman’s rho = 0.843, *p* < 0.01 for the non-judgmental attitude subscale; Spearman’s rho = 0.927, *p* < 0.001 for the refocusing subscale) indicate high semantic alignment with the original.

## STAGE 2

The preliminary version of the questionnaire translation was subjected to a validation study. The aim of the second stage of the research was to determine the internal structure of the questionnaire and to verify that the tool, just like the original version, arranges itself into a three-factor structure. For this purpose, stage 2 of the study was conducted, involving an exploratory factor analysis with a systematic analysis of the factor loadings for the individual items, and scale reliability was assessed using the Cronbach’s alpha internal consistency method.

### Participants

Participants were recruited using two distinct approaches: direct recruitment in sport clubs and online recruitment facilitated through sport clubs. A total of 335 athletes (130 females and 205 males) participated in the study (M_age_ = 20.40 ± 2.73 years). All the respondents surveyed were active athletes participating regularly in sports competitions. Considering their performance level, 22% of respondents competed at the international level, 35.8% at the national level, 18.8% at the regional level and 23% at the amateur level. The mean length of training experience of the respondents was 9.63 ± 5.21 (min = 2; max = 26). The largest group included soccer players (n = 103), followed by judokas (n = 40), swimmers (n = 36), volleyball players (n = 33), track and field athletes (n = 19), handball players (n = 10), badminton players (n = 8), basketball players (n = 9), modern pentathletes (n = 9), and others.

### Design and Procedures

Before completing the questionnaire, participants read the consent form, in which they were informed of the purpose of the study, the confidentiality of the data collected and the voluntariness of their participation. Most of the data were collected through direct contact with athletes, but some surveys were sent to respondents in other parts of Poland via an online form using Office Forms.

### Results of Stage 2

#### Factor Structure of the Mindfulness Inventory for Sport: Initial Version

Before testing the factor structure of the initial version of the MIS-PL, the discriminatory power coefficient of the test items was analyzed. The discriminatory power indices of all the items analyzed ranged from rbi = 0.31 to rbi = 0.53, thus the decision was made to include all the scale items analyzed in further statistical analyses.

#### Exploratory Factor Analysis

In order to determine the internal structure of the first version of the MIS-PL, the item set was subjected to an exploratory factor analysis procedure. The factor analysis was preceded by the Bartlett’s test of Sphericity (x^2^ = 833; df = 105; *p* < 0.001) and the KMO test for sampling adequacy (0.748), which confirmed the justification of the hypothesis concerning the existence of the scale’s factor structure, and therefore also the justification of conducting an exploratory factor analysis.

Maximum likelihood estimation with Promax rotation was used in the analysis, because according to the original Mindfulness Inventory for Sport tool, the subscales should be correlated with one another. A scree plot and the Kaiser criterion were used to determine the number of factors. It was assumed that the Polish version of the MIS, should also adopt a three-factor form (in line with the original version). The analysis of the scree plot and the analysis of the Kaiser values (eigenvalue greater than one; 1.69; 1.55 and 1.40, respectively) confirmed the justification of the procedure. The three-factor model explained 30.9% of the variance.

It was assumed that only items meeting the following two criteria would be included in the Polish version of the Mindfulness Inventory for Sport: 1) the factor loading should reach > 0.30 on one factor, and 2) the factor loading on the remaining factors should not exceed 0.30. The results obtained are presented in Table 1. The results obtained are consistent with those obtained in the research by Thienot et al. (2019), however, lower factor loading values were obtained for most of the items compared to the original MIS.

**Table 1:** Factor loadings for individual test items.

Items	Components		
	1	2	3
1. I am aware of the thoughts that are passing through my mind.			0.332
4. I am able to notice the intensity of nervousness in my body.			0.636
7. I am able to notice the sensations of excitement in my body.			0.588
10. I am able to notice the location of physical discomfort when I experience it.			0.674
13. I pay attention to the type of emotions I am feeling.			0.313
2. When I become aware that I am thinking about a past performance, I criticize myself for not being focused on my current performance.		0.580	
5. When I become aware that I am angry at myself for making a mistake, I criticize myself for having this reaction.		0.492	
8. When I become aware that I am not focusing on my own performance, I blame myself for being distracted.		0.547	
11. When I become aware that I am thinking about the final result, I blame myself for not being focused on relevant cues for my performance.		0.599	
14. When I become aware that I am really upset because I am losing, I criticize myself for reacting this way.		0.523	
3. When I become aware that some of my muscles are sore, I quickly refocus on what I have to do.	0.427		
6. When I become aware that I am thinking about how tired I am, I quickly bring my attention back to what I should focus on.	0.549		
9. When I become aware that I am really excited because I am winning, I stay focused on what I have to do.	0.517		
12. When I become aware that I am tense, I am able to quickly bring my attention back to what I should focus on.	0.645		
15. When I become aware that I am not focusing on my own performance, I am able to quickly refocus my attention on things that help me to perform well.	0.682		

*Note*. Extraction method: Exploratory factor analysis: maximum likelihood estimation with Promax rotation. Only factor loadings > 0.30 are displayed.

#### Reliability Analysis

In the subsequent step, the subscales extracted using exploratory factor analysis were subjected to reliability analysis. This was based on determining Cronbach’s alpha internal consistency coefficients, calculated separately for each factor extracted. All the questionnaire subscales had sufficient reliability coefficients: for factor 1, α = 0.70; for factor 2, α = 0.68; and for factor 3, α = 0.64. An additional analysis of the item-total correlation matrix showed that all items achieved values above the recommended 0.20 (Clark and Watson, 1995).

The results for the Cronbach’s α coefficient were analyzed after removal of the items from the scale (α-if-item-deleted). In the first subscale, the α coefficient would range from 0.61 to 0.69, in the second one, from 0.61 to 0.64, and in the third one, from 0.55 to 0.63. Analysis of the results showed that no deletion of any item from the questionnaire would improve the reliability of the test in a significant manner.

## STAGE 3

The initial version of the MIS-PL identified in the second stage of the research was subjected to a re-verification procedure of the internal structure of the questionnaire in order to be able to demonstrate the stability and repeatability of the factor structure of the scale. To achieve this, a confirmatory factor analysis (CFA) was performed using the combined generalized least squares and maximum likelihood estimation method. To evaluate the global model fit, fit indices including the Root Mean Square Error of Approximation (RMSEA; ≤ 0.06), the Standardized Root Mean Residual (SRMR; ≤ 0.08), the Comparative Fit Index (CFI; ≥ 0.90), the Tucker-Lewis Index (TLI; ≥ 0.90), the Goodness of Fit Index (GFI; ≥ 0.90) and the Adjusted Goodness of Fit Index (AGFI; ≥ 0.85) were calculated, based on recommendations by Schermelleh-Engel et al. (2003).

To further determine the validity of the Polish version of the tool, an analysis was performed of the correlation of the questionnaire’s subscales with the state of flow, worry, concentration disruption, conscious presence and mental toughness, making it possible to estimate the criterion validity of the MIS-PL.

Reliability analysis was performed by assessing the Cronbach’s alpha internal consistency coefficient and absolute stability for each of the subscales extracted. The test-retest method was used to assess the absolute stability of the test. Eight weeks after the first test, a re-test was performed using the same test (for a sample of 30 biathlon athletes).

All analyses were performed using Statistica 13.1 (StatSoft PL) and Jamovi Version 2.3.0.0 software.

## Participants

A total of 261 athletes (116 females and 145 males), recruited directly or online through sport clubs, participated in the study (M_age_ = 20.19 ± 2.41 years, min = 16, max = 36). All study participants actively took part in regular sports competitions, either at the amateur (n = 48), regional (n = 42), national (n = 127) or international level (n = 44). The largest group comprised soccer players (n = 56), biathletes (n = 33), swimmers (n = 32), volleyball players (n = 32) and other athletes (n = 19). The average training experience was M = 9.84 ± 3.59 (min = 4 and max = 26 years).

### Measures

#### Mindful Attention Awareness Scale (MAAS)

Mindfulness in daily life was measured using the Mindful Attention Awareness Scale (MAAS), consisting of 15 items, and used to measure trait mindfulness understood as the awareness of current events and experiences in daily life (Brown and Ryan, 2003). Items are rated on a 6-point Likert scale from “almost never” to “almost always”. The MAAS demonstrates satisfactory indices of reliability and validity (Brown and Ryan, 2003). The Polish versions of the scale (Radoń, 2014) were used in the study.

#### Dispositional Flow Scale 2 (DFS-2)

The DFS-2 (Jackson and Eklund, 2002) consists of 36 items and serves as a questionnaire for evaluating an individual’s inclination toward experiencing flow. It evaluates nine dimensions of flow: challenge-skill balance, action-awareness merging, clear goals, unambiguous feedback, concentration on the task, sense of control, loss of self-consciousness, transformation of time, and autotelic experience. By adding all scores, a global flow score can be derived, reflecting the individual’s disposition for experiencing flow. The study used the Polish version of the questionnaire, which is also characterized by satisfactory validity and reliability indicators (Józefowicz et al., 2022).

#### Sport Mental Toughness Questionnaire (SMTQ-P)

The SMTQ-P is a Polish version of the SMTQ (Sheard et al., 2009), which is used to measure mental toughness. The SMTQ-P consists of 13 items and differs slightly in structure from the original SMTQ questionnaire (Guszkowska et al., 2019). The SMTQ-P provides a global measure of mental toughness as well as four subscales: confidence (athletes' confidence in their own abilities to accomplish objectives and outperform others), effectiveness (reflecting determination and the ability to concentrate), emotional control (the belief in one's personal influence and capacity to achieve desired outcomes with specificity), and execution of tasks (reflecting individual responsibility and unyielding attitude).

#### Sport Anxiety Scale (SAS)

The SAS (Smith et al., 1990) comprises 21 items designed to assess the level of anxiety experienced by athletes before or during competition. Participants rate items on a 4-point scale ranging from 1 (not at all) to 4 (very much so). The SAS yields a total score and three subscale scores: somatic anxiety, worrying, and concentration disruption. The SAS has satisfactory indices of test-retest reliability, internal consistency, and construct and convergent validity (Smith et al., 1990). The study used two subscales (worry and concentration disruption) of the Polish version of the SAS adapted by Krawczyński (Stankiewicz, 1996).

#### Procedure

The athletes surveyed completed a packet of questionnaires including: the Polish version of the MIS developed in the first and second stages of the study and the MAAS, DFS-2, SMTQ-P and SAS questionnaires. Before completing the questionnaires, respondents were informed about the nature and the purpose of the research, their right to withdraw from it, the confidentiality of the data collected and their voluntary consent to participate in the research. Again, most of the data were collected through direct contact with athletes. The questionnaire package, together with the relevant information concerning the research, was also collected online using Office Forms.

### Results of Stage 3

#### Factor Structure of the Mindfulness Inventory for Sport

Descriptive statistics for each scale item are shown in Table 2.

**Table 2:** Descriptive Statistics for the MIS-PL 15 items.

Descriptives						
Item	Mean	Median	Standard deviation	Skewness	Kurtosis	Shapiro-Wilk W
1	4.85	5	0.940	−0.668	0.266	0.863***
4	4.53	5	1.12	−0.608	0.187	0.896***
7	4.92	5	1.06	−0.917	0.462	0.845***
10	4.85	5	1.08	−0.918	0.686	0.854***
13	4.49	5	1.23	−0.686	0.066	0.892***
2	3.30	3	1.31	−0.045	−0.808	0.932***
5	3.63	4	1.51	−0.034	−0.926	0.930***
8	3.92	4	1.27	−0.135	−0.581	0.932***
11	3.74	4	1.32	−0.181	−0.480	0.935***
14	3.35	3	1.39	0.161	−0.744	0.935***
3	4.50	5	1.16	−0.640	0.138	0.897***
6	4.24	4	1.15	−0.399	−0.096	0.917***
9	4.68	5	1.02	−0.575	−0.087	0.882***
12	3.88	4	1.08	−0.136	−0.217	0.922***
15	4.16	4	1.13	−0.443	0.137	0.912***

* *p* < 0.05; ** *p* < 0.01; *** *p* < 0.001

#### Confirmatory Factor Analysis

To confirm the stability of the questionnaire’s internal structure, a confirmatory factor analysis was performed using the combined generalized least squares and maximum likelihood estimation method.

The absolute fit indices obtained, i.e., x^2^(87) = 156, *p* < 0.001, RMSEA= 0.05 (90% CI 0.04–0.07), SRMR = 0.05 indicated a good level of the model fit. Furthermore, additional fit indices such as GFI = 0.92, AGFI = 0.89 and RMS = 0.06 also indicated an acceptable fit of the data to the model (Schermelleh-Engel et al., 2003). The CFI and TLI indices did not achieve the assumed minimum values, but the respective values of 0.89 and 0.87 indicated marginal deviations from an adequate fit ( > 0.90).

Correlation matrix analysis showed that the awareness and refocusing subscales were positively and significantly correlated with one another (r = 0.38, *p* < 0.001). The remaining correlations among the MIS-PL subscales were non-significant. This was consistent with the results obtained using the original tool (Thienot et al., 2019).

#### External Aspects of Construct Validity

Correlations among the three MIS-PL subscales and other variables were used to verify external aspects of validity of the MIS-PL (convergent validity). Table 3 presents the results.

#### Trait Mindfulness in Daily Life

In line with results obtained so far (Thienot et al., 2019; Wieczorek et al., 2022), it was assumed that two MIS-PL subscales: non-judgmental and refocusing would be positively correlated with the tendency to be mindful in daily life. The theoretically expected correlations between the refocusing subscale and the MAAS (r = 0.227, *p* < 0.001), as well as the non-judgmental subscale and the MAAS (r = 0.238, *p* < 0.001) were confirmed. No positive correlation was obtained between the awareness subscale and the MAAS (r = 0.110, *p* = 0.076), which was also consistent with results obtained in the original (Thienot et al., 2019) and the German version of the MIS (Wieczorek et al., 2022).

#### Worry and Concentration Disruption

Bishop et al. (2004) identified two aspects of mindfulness: attention self-regulation and adopting an accepting stance toward experiences. They assumed that the refocusing subscale would negatively correlate with worry and concentration disruption, while the non-judgmental subscale would particularly correlate negatively with worry. As expected, a negative correlation between the MIS-PL refocusing and worry (*r* = −0.309, *p* < 0.001), refocusing and concentration disruption (*r* = −0.314, *p* < 0.001), non-judgmental and worry (*r* = −0.250, *p* < 0.001), and non-judgmental and concentration disruption (*r* = −0.129, *p* = 0.038) could be identified. No significant correlation was obtained between the MIS-PL awareness subscale and worry (*r* = −0.079, *p* = 0.205). A negative correlation of weak strength (*r* = −0.173, *p* = 0.005) was obtained between awareness and concentration disruption. Our results were consistent with those of Thienot et al. (2019).

#### Flow

According to the existing state of knowledge, mindfulness is related to dispositional flow, especially aspects such as action and awareness, clear goals, balance between challenge and skill, explicit feedback, and focus on the task (Chen and Meggs, 2021; Glass et al., 2018; Kaufman et al., 2009; Kee and Wang, 2008).

Most of the assumed correlations between the MIS-PL subscales and the flow dimensions were confirmed (Table 3). Similarly to the study by Thienot et al. (2019), significant positive correlations with the overall dispositional flow index were obtained only in the awareness (*r* = 0.282, *p* < 0.001) and refocusing (*r* = 0.482, *p* < 0.001) dimensions. Also in most of the remaining flow dimensions, positive correlations were obtained with two subscales of the MIS-PL: awareness and refocusing. The transformation of time dimension of flow was not significantly associated with any of the MIS-PL subscales. No positive correlations were obtained either between attentiveness and the action and awareness dimension of flow. The weak negative correlation obtained between non-judgmental and action and awareness (*r* = −0.139, *p* < 0.024) was surprising and opposite to the hypotheses assumed.

**Table 3:** Correlations between the MIS-PL (Polish version) and the sub scales of conceptually related constructs.

	*Mindfulness Inventory for Sport - PL*		
	Awareness	Non-judgmental	Refocusing
***Mindfulness Inventory for Sport - PL***			
Awareness			
Non-judgmental	−0.10		
Refocusing	0.38***	0.02	
***Mindful Attention Awareness Scale***			
Mindfulness	0.11	0.24***	0.23***
***Sport Anxiety Scale***			
Worry	−0.08	−0.25***	−0.31***
Concentration Disruption	−0.17**	−0.13*	−0.31***
***Dispositional Flow Scale 2***			
Flow disposition	0.28***	0.05	0.48***
Challenge-skill balance	0.29***	0.12	0.45***
Action and awareness	0.11	-0.14*	0.08
Clear goals	0.28***	-0.04	0.43***
Unambiguous feedback	0.28***	0.06	0.41***
Task concentration	0.14*	0.04	0.37***
Sense of control	0.32***	0.08	0.45***
Loss of self-consciousness	0.04	0.15*	0.16**
Transformation of time	0.06	−0.07	0.09
Autotelic experience	0.18**	0.02	0.41***
***Sport Mental Toughness Questionnaire***			
Mental toughness	0.22***	0.19**	0.36***
Confidence	0.24***	0.12	0.31***
Effectiveness	0.15*	0.20**	0.35***
Emotional control	0.07	0.26***	0.20**
Execution of tasks	0.13*	−0.03	0.14*

n = 261; * *p* < 0.05; ** *p* < 0.01; *** *p* < 0.001

#### Mental Toughness

Another variable that seems to be related to mindfulness in sports is mental toughness (Rintaugu et al., 2022), thus based on previous research, it was hypothesized that the MIS-PL subscales should positively correlate with mental resilience, in particular emotional control (Kaufman et al., 2018; Kee and Wang, 2008; Minkler et al., 2020). The results confirmed the original assumptions: all the MIS-PL subscales correlated significantly positively with mental toughness (*r* = 0.217, *p* < 0.001 for awareness; *r* = 0.190, *p* = 0.002 for non-judgmental; *r* = 0.364, *p* < 0.001 for refocusing). In addition, positive correlations were observed between the awareness subscale and self-confidence (*r* = 0.239, *p* < 0.001), effectiveness (*r* = 0.146, *p* = 0.018), and task execution (*r* = 0.129, *p* = 0.037). The non-judgmental subscale correlated positively with effectiveness (*r* = 0.196, *p* < 0.001) and emotional control (*r* = 0.255, *p* < 0.001). The refocusing subscale, in turn, correlated with confidence (*r* = 0.311, *p* < 0.001), effectiveness (*r* = 0.347, *p* < 0.001), emotional control, (*r* = 0.197, *p* < 0.001), and task execution (*r* = 0.137, *p* = 0.026).

#### Reliability Analysis

The reliability of the Polish version of the MIS-PL was analyzed once again. The Cronbach’s alpha internal reliability coefficient was calculated separately for each subscale distinguished. Also that time, all the subscales of the questionnaire demonstrated adequate reliability indices: for awareness, α = 0.65; for refocusing, α = 0.70; for non-judgmental, α = 0.69, and were similar to those obtained in the second stage of the research. Further analysis of Cronbach’s α coefficients after removing items from the scale (α-if-item-deleted) showed that deletion of any item in the questionnaire would not significantly improve the test’s reliability.

The test-retest method was also used to estimate the test’s absolute stability. For this purpose, re-measurement using the MIS-PL was performed after 8 weeks with 30 biathlon athletes. Correlation analysis between the first and the second test results found that the absolute stability of the MIS-PL was high, ranging from 0.77 for refocusing to 0.79 for non-judgmental and 0.87 for awareness.

## Discussion

The aim of the current study was to examine the psychometric properties of the Polish version of the Mindfulness in Sport scale. As a result of the statistical analyses performed, a three-factor structure was confirmed in the Polish version (MIS-PL), with the following dimensions: 1) awareness, 2) non-judgmental, and 3) refocusing. A two-stage verification of the structure confirmed its stability. The analyses of the test items performed in the second stage of the research confirmed the adequacy of the psychometric properties of each item in relation to the relevant subscale. The absolute fit indices obtained during the CFA indicated a good fit of the model. The model fit analyses were extended to include verification of the Goodness of Fit Index (GFI = 0.92) and the Adjusted Goodness of Fit Index (AGFI = 0.89), which also showed an acceptable level of fit (Schermelleh-Engel et al., 2003). Neither the Comparative Fit Index (CFI) nor the Tucker-Lewis Index (TLI) reached the minimum values indicating the adequate model fit (Schermelleh-Engel et al., 2003), but the values obtained were similar to those obtained in the German version of the MIS (Wieczorek et al., 2022). The MIS-PL subscales rather formed separate dimensions, as only awareness and refocusing subscales correlated positively with each other, which is consistent with the original version of the MIS, and also confirms the relative internal stability of the tool (Thienot et al., 2019).

To assess internal consistency, the Cronbach alpha formula was used. Only the refocusing subscale achieved the minimum value (0.70) generally considered acceptable (Clark and Watson, 1995; Nunnally and Bernstein, 1994). The reliability of the remaining subscales deviated marginally from the accepted cut-off value. However, bearing in mind that the alpha coefficient depends on the length of the test (the number of items each subscale is composed of), the necessary additional item-total correlation matrix analysis was performed. All items achieved values above the recommended 0.20, which makes it possible, despite the lower Cronbach’s alpha indices compared to the original version, to consider the MIS-PL a tool with acceptable reliability (Clark and Watson, 1995). In addition, the analysis of the absolute stability of the Polish version of the MIS was extended to include the test-retest method. The indices obtained confirmed high absolute stability of the MIS-PL, which also extends the analyses of reliability of the original tool by Thienot et al. (2019).

To further assess the validity of the MIS-PL, the awareness, non-judgmental, and refocusing subscales were correlated with conceptually associated constructs, assuming significant relationships between them. The study sought to use the same constructs as in the original version of the MIS scale, but due to the lack of appropriate adaptations of the tools in the Polish language, this was not fully possible. Consequently, only the associations of the MIS-PL subscales with trait mindfulness in daily life, worry and concentration disruption, and trait flow were verified. Mindfulness-based interventions on a group of athletes in a number of studies led to increased mindfulness also in daily life (Noetel et al., 2019), providing reasons to assume that the MIS would be positively related to the global MAAS score. As expected, positive correlations were obtained between mindfulness in daily life and the non-judgmental and refocusing subscales. The lack of significant correlations between the awareness subscale and the MAAS may be due to the different contents of awareness on which these tools focus, as already pointed out by Wieczorek et al. (2022). In the MIS, the items concern primarily the awareness of one’s own body and of the emotions experienced, while in the MAAS, they mainly concern awareness in everyday life.

Previous studies have also shown that MBIs have a small to medium effect on competitive anxiety (Ong and Chua, 2021). Observational designs have also shown an association between mindfulness and anxiety (Noetel et al., 2019), hence it was assumed that the MIS-PL subscales should be negatively associated with worry and concentration disruption. The associations between the MIS and cognitive anxiety were also demonstrated in earlier MIS adaptations (Wieczorek et al., 2022). The results of our research have proven to be in line with the assumed direction.

The association of mindfulness with the state of flow, as well as with the trait of flow, has been confirmed in many studies, both experimental, verifying the effectiveness of MBIs in sports, and correlational ones, and the effect can be considered strong (Noetel et al., 2019). The analyses of the research on the Polish version of the MIS included an assessment of the associations between the sub-dimensions of the trait of flow and the MIS-PL subscales, which also significantly extended the verification of the above tool. Consequently, it was hypothesized that all dimensions of mindfulness assessed by the MIS scale would exhibit significant, positive associations with the dimensions of flow. As assumed on the basis of previous studies (Chen and Meggs, 2021; Glass et al., 2018; Kaufman et al., 2009; Kee and Wang, 2008), positive correlations of the MIS were obtained with almost the majority of flow dimensions. Only the non-judgmental subscale was not significantly associated with the state of flow and its sub-dimensions, which is consistent with the results of Thienot et al. (2019). However, bearing in mind the previous reports on mindfulness in sport, the validity assessment was extended to verify the relationship between mindfulness and mental resilience. The findings confirm that emotional control is associated with attentiveness, which is supported by both the theoretical grounds (Kaufman et al., 2018) and the research results obtained (Kee and Wang, 2008, Minkler et al., 2020). In turn, the relationship between self-confidence and mindfulness has so far only been confirmed in few studies (Mehrsafar et al., 2019; Oguntuase and Sun, 2022; Thompson et al., 2011), and the relationship with mental toughness was only confirmed in the study by Rafeeque and Sultan (2016) and Wu et al. (2021). Therefore, the findings of the above research make it possible to expand knowledge concerning the importance of mindfulness in sports.

In conclusion, the results of the statistical analyses show that the MIS-PL can be considered a tool with satisfactory validity and reliability. During the process of creating the Polish version of the scale, an effort was made to maintain similarity in the way of testing reliability and validity and in the interpretation of the results obtained in order to maintain face, functional, and psychometric equivalence, as well as faithfulness of translation and reconstruction to the original scale. However, it was not entirely possible to maintain complete faithfulness to the scale creation process as in the original version of the MIS. Therefore, despite the satisfactory results obtained, further analyses and verification of the psychometric properties of the Polish version of the MIS are still necessary.

## Limitations and Further Research

In future research, it will be essential to verify once again the reliability of the MIS-PL subscales. It would also be worthwhile to extend the criterion validity analysis to verify the associations of the MIS-PL subscales with perfectionism and rumination. In addition, due to the insufficient number of respondents at the stage of division into groups, it was not decided to verify the stability of construct validity depending on gender and the type of sport played. This is why further research is nevertheless required to verify factorial validity and to confirm the stability of the MIS structure in the Polish version, taking into account gender and the type of sport played (individual vs. team). Moreover, the verification of the internal validity of the Polish version is necessary, given that the values of CFI and TLI indicators showed marginal deviations from an adequate fit (≥0.90). To sum up, it will be crucial to re-evaluate the reliability of MIS-PL subscales and assess construct validity stability across gender and types of sports.

## Conclusions

The strengths of the study include comprehensive validation of the MIS-PL, confirming its reliability and validity for assessing mindfulness in Polish athletes. This not only enhances research accuracy, but also offers a culturally adapted tool for practitioners to effectively measure and address mindfulness in sports contexts. The validation process of the MIS-PL was expanded to include assessments of absolute stability, as well as its associations with mental toughness and all dimensions of flow, thereby enhancing comprehension of the interplay between mindfulness in sport and athletes' performance quality during competitive sports events.
